# Molecular identification and phylogenetic analysis of *Lactobacillus* and Bifidobacterium spp. isolated from gut of honeybees (*Apis mellifera*) from West Azerbaijan, Iran

**Published:** 2016-12-15

**Authors:** Mohammad Farouq Sharifpour, Karim Mardani, Abdulghaffar Ownagh

**Affiliations:** 1 Private Veterinary Practitioner, Mahabad, Iran; 2 Department of Food Hygiene and Quality Control, Faculty of Veterinary Medicine, Urmia University, Urmia, Iran; 3 Department of Microbiology, Faculty of Veterinary Medicine, Urmia University, Urmia, Iran

**Keywords:** *Apis mellifera*, Honeybee, Lactic acid bacteria, Molecular identification, Phylogenetic analysis

## Abstract

Polymerase chain reaction and restriction fragment length polymorphism (PCR-RFLP) and phylogenetic analysis were used for molecular identification of lactic acid bacteria (LABs) isolated from *Apis mellifera*. Eighteen honeybee workers were collected from three different apiaries in West Azerbaijan. LABs from the gut of honeybees were isolated and cultured using routine biochemical procedures. Genomic DNA was extracted from LABs and a fragment of 1540 bp in size of 16S rRNA gene was amplified. PCR products were digested using *Hinf*I endonuclease and digested products with different RFLP patterns were subjected to nucleotide sequencing and phylogenetic analysis. The results revealed that *Lactobacillus* and *Bifidobacteria* spp. are were the most abundant LABs in honeybee gut. Phylogenetic analysis showed that both *Lactobacillus* and *Bifidobacterium* were closely clustered with high similarity percentage with the same bacteria isolated from honeybees’ gut elsewhere. It was concluded that LABs isolated from honeybees had low sequence divergence in comparison with LABs isolated from other sources such as dairy products.

## Introduction

Lactobacillus species are considered as the most important and also dominant genus of lactic acid bacteria (LAB) found commensally in honeybee gut and also human and intestines of other animals.^1^^,^^2^ As probiotics, Lactobacillus together with Bifidobacterium could play a key role in the promotion of animal and human health.^3^^,^^4^ Due to several investigations Lactobacilli have significant effects on prevention and treatment of various human gastrointestinal disorders, infectious enteritides and enterocolitides, besides enteric and colorectal cancers.^5^^-^^7^ In domestic ruminants these bacteria play an important role in improving the nutritional efficiency.^8^^-^^10^

In honeybees’ gastrointestinal tract, Lactobacilli are dominant LAB. With 183 species, Lactobacillus is one of the most important genus of beneficial bacterial population,^2^^,^^11^ acting as immuno-stimulators and the first frontier of fermentation.^12^^,^^13^

Honey is a liquid substance made up in the honey-stomach of honeybees from collected nectar and pollen by foraging workers. Honey bears various therapeutic properties, antibiotic and wound healing effects.^14^ These properties are considerably due to the chemical compounds inside honey, e.g. hydrogen peroxide, flavonoid and phenolic acids,^15^ and a considerable contents of unidentified component with antibacterial effects.^16^

Honeybee larvae gut may be occupied by LABs through various routes including ingested pollen, and other floral matter, dust, and the honeybees’ gastrointestinal tract secretions in the hive before pupation.^17^ Honey stomach is an expanded anatomical part of honeybee gut, developed from esophagus or honey crop.^2^ The organ provides suitable conditions (i.e. micro-aerobic state and 35 ˚C temperature) for optimal growth of LABs when it is filled with nutrients and nectar.^18^

Cultivation and 16S rRNA sequence analysis used for molecular characterization of the mid- and hindgut microbiota of the honeybee Apis mellifera, showed the existence of only eight bacterial phylotypes^19^^,^^20^ including Lactobacillus and Bifidobacterium spp.^21^^-^^23^

The aim of the present study was to characterize Lactobacilli and Bifidobacteria from gastrointestinal tract of honeybee from West Azerbaijan, Iran using molecular methods and comparing them with other microbiota isolated from honeybees from different parts of the world.

## Materials and Methods

Sample collection. A total number of 18 individual worker honeybees were collected in summer from three geographically distant apiaries in West Azerbaijan province of Iran. The honeybees were brought to laboratory alive and then anesthetized with ether to handle easier while the whole intestinal tracts, esophagus to rectum were aseptically handled to avoid contamination with the external surface of the bee body.^2^

Isolation of **Lactobacilli** and **Bifidobacteria. **The gastrointestinal content of each honeybee was mixed in sterile phosphate buffer, made a suspension to subsequent bacterial culture. Prepared suspensions were cultivated in De Man, Rogosa and Sharpe (MRS) agar (Merck, Darmstadt, Germany) twice, under anaerobic condition using Anaerocult^®^ C-Merck Millipore (Merck) for four days at 35 ˚C. Then grown bacterial colonies were transferred to MRS broth and cultivated under the same condition for another four days using an initial screening of Lactobacilli and Bifidobacteria, gram-positive and catalase-negative bacilli were chosen.^2^^,^^24^^-^^26^ The isolates were maintained as frozen stocks at –20 ˚C in MRS broth supplemented with 15% (v/v) glycerol for further analysis.^2^

Extraction of bacterial genomic DNA. Genomic DNA from cultivated Lactobacilli and Bifidobacteria were extracted using a rapid salt-extraction method described by Aljanabi and Martinez.^27^ Briefly, bacterial cells from 1.5 mL of 18 to 24 hr culture in MRS broth were spun down and re-suspended in 400 µL of homogenizer buffer and the extraction procedure was followed as described previously.

Polymerase chain reaction. A fragment of 1540 bp in size from 16S rRNA gene was amplified using a pair of general primers EGE1: (5′-AGAGTTTGATCCTGGCTCAG-3′) and EGE2: (5′-CTACGGCTACCTTGTTACGA-3′).^28^ The PCR was performed in 25 µL reaction volume, containing 0.5 µL Taq DNA polymerase (5 U per µL), 1 µL MgCl_2_ (50 mM), 0.5 µM of each primer, 50 µM each of dATP, dCTP, dGTP and dTTP, 2.5 µL of 10X PCR buffer and 100 ng of genomic DNA. Thermal conditions for PCR were as follow: Initial denaturation at 94 ˚C for 5 min, followed by 40 cycles of denaturation at 94 ˚C for 1 min, annealing at 56 ˚C for 1 min, elongation at 72 ˚C for 1 min, and a 10 min final elongation at 72 ˚C.^28^ PCR products were electrophoresed in 1.5% agarose gel and visualized by UV transilluminator (Synoptics, Cambridge, UK) after staining with ethidium bromide.

Purificationandrestrictionendonucleasedigestion of PCR products. The PCR products were purified using AmbiClin Kit (Vivantis, Subang Jaya, Malaysia). Purified products were digested using HinfI endonuclease (Vivantis). The digestion reaction was performed in 15 µL reaction volume, containing 1 µL HinfI, 1.5 µL reaction Buffer, 7.5 µL dH_2_O and 5 µL of purified PCR product. The reaction mixture was incubated in 37 ˚C for 4 hr then the digested PCR products were electrophoresed, stained with ethidium bromide and visualized via UV transilluminator (Synoptics).

DNA sequencing and analyses. The PCR products of four bacterial isolates with different RFLP patterns were chosen for sequencing. Purified PCR products were sent to SinaClon Company (Tehran, Iran) for sequencing. Obtained nucleotide sequences of 16S rRNA were searched against GenBank (National Centre for Biotechnology Information, Rockville Pike, Bethesda, USA) using the advanced BLAST similarity search option and compared to the 16S rRNA sequences of Lactobacilli and Bifidobacteria strains from GenBank (Table 1). Nucleotide sequences were aligned and compared to other nucleotide sequences from GenBank using Clustal W and phylogenetic tree was generated using the neighbor-joining method in MEGA software (version 6.0; Biodesign Institute, Tempe, USA).^29^^,^^30^

**Table 1:** Primer sequences used for qRT-PCR

	**Sequence**	**Origin of isolation**	**Accession code**
***Lactobacilli***	***Lactobacillus insectis*** ** strain 2L1**	Honeybee gut	AY667699
	***Lactobacillus kunkeei*** ** strain 93-30**	Honeybee gut	JQ009345
	***Lactobacillus kunkeei*** ** strain B8_7LCO2**	Honeybee gut	KF600484
	***Lactobacillus kunkeei*** ** strain G5_13_3MO2**	Honeybee gut	KF600202
	***Lactobacillus kunkeei*** ** strain H14_5_1BCO2**	Honeybee gut	KF599427
	***Lactobacillus kunkeei*** ** strain H19_1_1_2BCO2**	Honeybee gut	KF599431
	***Lactobacillus kunkeei*** ** strain H19_5_1TCO2**	Honeybee gut	KF599370
	***Lactobacillus sp.*** ** 1F1**	Honeybee gut	AY667701
	***Lactobacillus sp.*** ** AcjLac1**	Honeybee gut	AB810023
	***Lactobacillus sp.*** ** Bma5**	Honeybee gut	EF187242
	***Lactobacillus sp.*** ** DAT823**	Honeybee gut	AB777211
	***Lactobacillus sp.*** ** G7_8_4CO2**	Honeybee gut	KF600368
	***Lactobacillus sp.*** ** H7_5_1MCO2**	Honeybee gut	KF599228
	***Lactobacillus sp.*** ** H8_4_2MCO2**	Honeybee gut	KF599241
	***Lactobacillus sp.*** ** H8_9_5MCO2**	Honeybee gut	KF599258
	***Lactobacillus sp.*** ** H8_12_5MO2**	Honeybee gut	KF599239
	***Lactobacillus sp.*** ** Hma2**	Honeybee gut	EF187240
	***Lactobacillus sp.*** ** Hma8N**	Honeybee gut	JX099551
	***Lactobacillus casei *** **strain MRTL1**	Milk	KC456363
	***Lactobacillus curvatus *** **strain BMG 157**	Meat	EU081014
	***Lactobacillus fermentum***	Human feces	AB932537
	***Lactobacillus paracasei subsp. paracasei *** **strain L3C21M6**	Cheese	KM096826
	***Lactobacillus plantarum *** **strain BMG 112**	Meat	EU081011
	***Lactobacillus plantarum *** **strain Lact09**	Cheese	FJ905313
	***Lactobacillus reuteri *** **strain NT09**	Human feces	JN813102
	***Lactobacillus sakei *** **strain BMG 126**	Meat	EU081017
	***Lactobacillus salivarius *** **strain AF-7**	Human feces	KT371516
	***Lactobacillus sp. *** **LMK3**	Cheese	AJ251560
	***Lactobacillus sp. *** **MSUGMIR-3**	Milk	JN561696
***Bifidobacteria***	***Bifidobacterium animalis subsp. lactis *** **strain S7**	Cheese	KJ463393
	***Bifidobacterium asteroides *** **PRL2011**	Honeybee gut	NR_102860
	***Bifidobacterium asteroides *** **strain Mbobb2t12**	Honeybee gut	HM534830
	***Bifidobacterium asteroides *** **strain JCM 8230**	Honeybee gut	LC071851
	***Bifidobacterium asteroides***	Honeybee gut	AB437355
	***Bifidobacterium crudilactis *** **strain FR62,b,3**	Milk	NR_115342
	***Bifidobacterium crudilactis *** **strain S10**	Cheese	KJ463394
	***Bifidobacterium crudilactis *** **strain S17**	Cheese	KJ463396
	***Bifidobacterium mongoliense***	Fermented milk	AB433856
	***Bifidobacterium sp. *** **Acbbto5**	Honeybee gut	HM534825
	***Bifidobacterium sp. *** **Afpor3**	Honeybee gut	HM534818
	***Bifidobacterium sp. *** **CU3-7**	Human feces	KF990498
	***Bifidobacterium sp. *** **FR59,b,2**	Milk	AY952448
	***Bifidobacterium sp. *** **Thsr10**	Honeybee gut	HM534826
	***Bifidobacterium Uncultured sp. *** **clone pAJ207**	Honeybee gut	AY370184

## Results

**Screening and phenotypic characterization of** ***Lactobacilli*** **and** ***Bifidobacteria*** **. **Different types of colonies developed on the surface of MRS agar plate after 24 to 48 hr of incubation at 37 ˚C. A number of 54 MRS agar plates were screened for small, round, opaque and white colonies and among them 27 bacterial colonies were purely isolated and showed biochemical characteristic of *Lactobacilli* and *Bifidobacteria*: Non-sporulated, cocci or rods gram-positive, non-motile cells, catalase negative and nitrate negative.

**Fig. 1:**
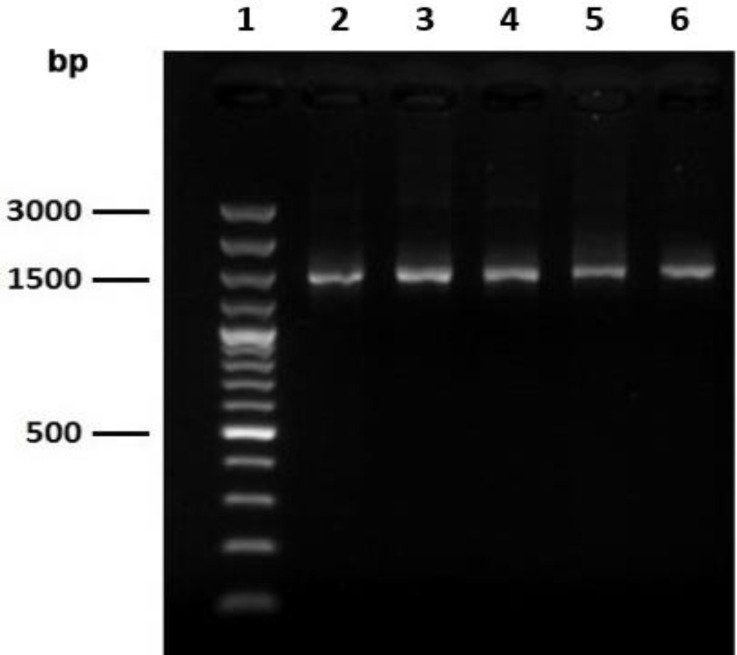
Amplification of 16S rRNA gene of bacteria isolated from honeybee. **Lane 1:** Molecular marker 50 bp (Vivantis, Subang Jaya, Malaysia); **Lanes 2-6:** PCR products from bacterial isolates

**Fig. 2:**
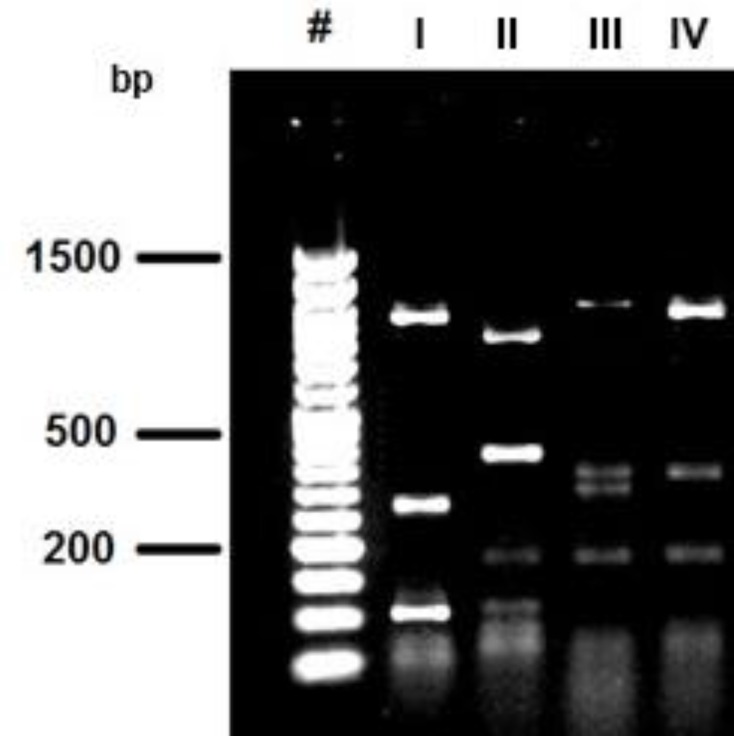
RFLP pattern of 16S rRNA gene of isolated bacteria from honeybee. **Lane #:** Molecular marker 50 bp (Vivantis); **Lanes I-IV:** four different RFLP patterns generated from 27 PCR products of bacterial isolates

**PCR and restriction fragment length polymorphism (RFLP). **Genomic DNA was extracted from all 27 bacterial colonies after incubating them in 5 mL MRS broth. A DNA fragment of 1540 bp in size was amplified for all isolates from three apiaries (Fig. 1). RFLP analysis of the PCR products revealed four different digestion patterns (Patterns I to IV) with *Hinf*I (Fig. 2). Out of 27 bacterial isolates, 15 (55.5%), 4 (14.8%), 5 (18.5) and 3 (11.1%) isolates generated RFLP pattern I, II, III and IV, respectively.

**Fig. 3.**
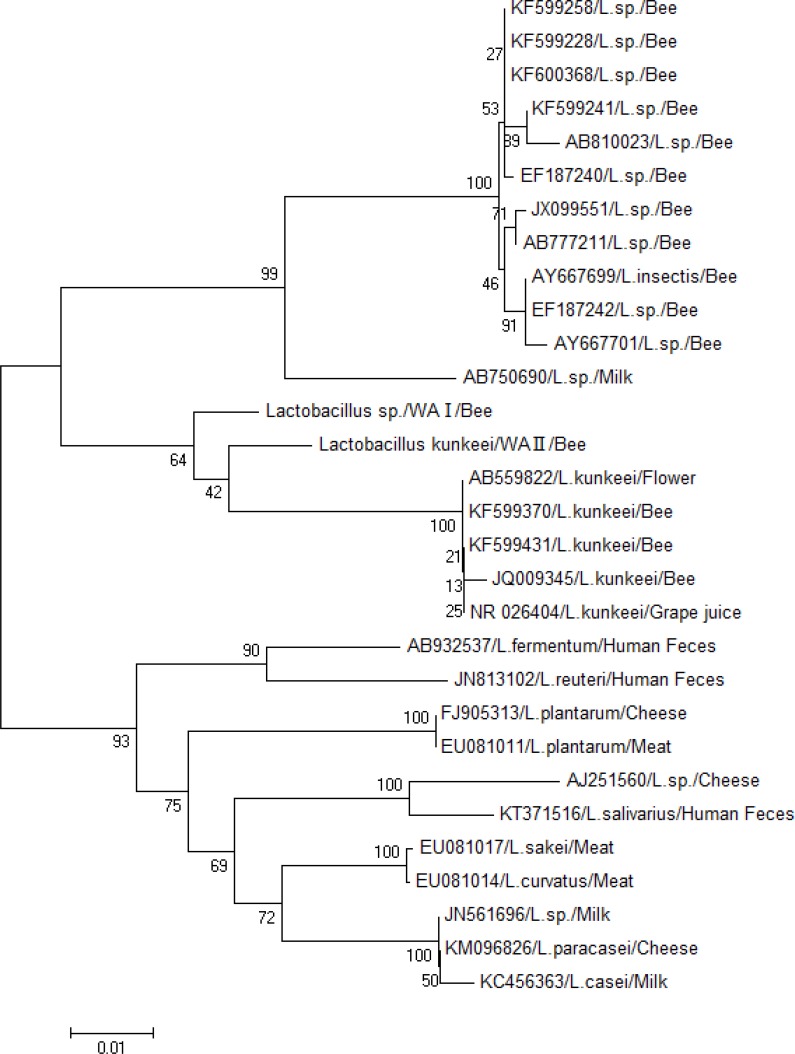
The evolutionary history was inferred using the Neighbor-joining method. The percentage of replicate trees in which the associated taxa clustered together in the bootstrap test (1000 replicates) are shown next to the branches.^39^ The tree is drawn to scale, with branch lengths in the same units as those of the evolutionary distances used to infer the phylogenetic tree. The analysis involved 30 nucleotide sequences. All positions containing gaps and missing data were eliminated. There were a total of positions in the final dataset. Evolutionary analyses were conducted in MEGA 6*.*0

**Phylogenetic Analysis. **Phylogenetic tree constructed based on neighbor-joining analysis of 16S rRNA gene revealed that *Lactobacilli* with RFLP patterns I and II were clustered with other *Lactobacilli *from honeybees and other sources. Comparison of the 16S rRNA gene sequence of *Lactobacilli *isolates from the present study with the corresponding *Lactobacillus* sequences from the GenBank database showed that *Lactobacilli* strains were placed in the evolutionary clade of *Lactobacillus* especially with honeybee origin. *Lactobacillus kunkeei* and *Lactobacillus *spp. were the dominated *Lactobacilli* from the gut of honeybees in West Azerbaijan. 16S rRNA nucleotide sequence BLAST showed high similarity (99.0 to 98.0 %) of isolated *Lactobacilli *in the present study with other *Lactobacilli *reported from honeybees elsewhere (Fig. 3).

**Fig. 4:**
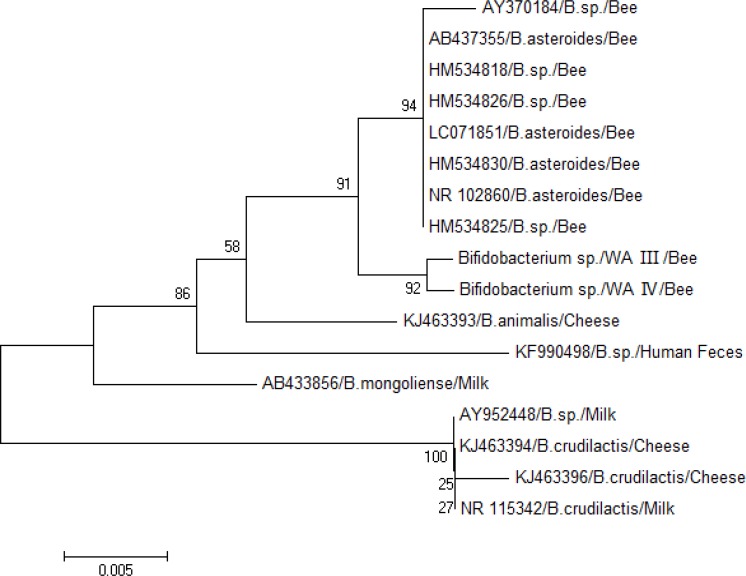
Phylogenetic tree of 16S rRNA gene of gastrointestinal Bifidobacteria spp. isolates from West Azerbaijan and other countries generated using neighbor-joining method in MEGA 6.0

Lactic acid bacteria with RFLP patterns III and IV were clustered with *Bifidobacteria*. The 16S rRNA gene phylogeny of the *Bifidobacteria* and related sequences from honeybees and dairy products showed that *Bifidobacteria* isolated from honeybees in the present study were more closely related to *Bifidobacteria* isolated from honeybees elsewhere (Fig. 4). Phylogenetic analysis results of this study showed that *Lactobacillus *and *Bifidobacteria* spp. were two dominant phylotypes in honeybee gut from West Azerbaijan.

## Discussion

 The majority of bacterial flora existing in honeybee’s gut are LABs.^31^ Because of the utilization of LABs as probiotics, their identification and characterization is of great importance. Due to biochemical similarity among LABs, molecular assays are the most powerful and accurate techniques for differentiation and characterization of LABs than traditional methods.^32^ There were limited number of reports regarding to the molecular characterization of *Lactobacillus *and *Bifidobacterium* spp. in honeybees until recent years. Nowadays, there are several reports on microbial diversity of honeybee gut using new molecular methods such as nucleotide sequencing, real time PCR and phylogenetic analysis.^33^^-^^36^ In the present study *Lactobacilli* and *Bifidobacteria* spp. were isolated from the gut of honeybee of West Azerbaijan, Iran and they were differentiated using PCR-RFLP and phylogenetic analysis based on 16S rRNA gene.

We showed that there were different *lactobacillus* and *Bifidobacteria* spp. in honeybee gut that could be differentiated using PCR-RFLP and nucleotide sequence analysis. The presence of two potentially LABs in honey-bee gut deserve to pay attention to microflora of honeybee digestive tract. The LABs are beneficial for humans and animals and presumably for honeybees as they produce antibacterial and antiviral compounds as organic acids, hydrogen peroxide, diacetyl, benzoate, and bacteriocins.^37^

The precise identification of LABs by phenotypic methods is difficult as it requires about 17 phenotypic tests to identify an isolate of LAB at the species level.^38^ Therefore, identification of microorganisms presenting probiotic properties with nutritional and economic importance is a prerequisite to select new strains among several bacterial isolates. Restriction profiling of 16S-23S rRNA has been successfully used for identification of the species level of *Lactobacillus *isolated from different sources.^40^ This technique was easily able to differentiate 45 new strains of *Lactobacillus* obtained from animals, human or foods. Ellegaard, *et al*.^34^ showed extensive intra-phylotype diversity in *lactobacilli* and *bifidobactreia *from the honeybee gut using genome sequencing and phylogenetic analysis of these bacteria.

The results of the present study revealed that the majority of LABs isolated from honeybee gut mainly belonged to genera *Lactobacillus* and *Bifidobacterium. *Similar findings were also reported by other researchers in which *Lactobacillus* and *Bifidobacterium* spp. found to be the majority bacteria isolated from digestive tract of other honeybees including *Apis mellifera*,^33^ *Apis dorsata*,^41^ and *Apiscerana*.^42^

The *Lactobacillus* and *Bfidobacteria* spp. isolated form honeybee in the present study were clustered with the same bacteria isolated from honeybee elsewhere with low sequence divergence, however, with distinct distance from with *Lactobacillus* and*Bifidobacteria* isolated from other sources such as dairy products. The low sequence divergence levels at 16S rRNA among LABs isolated from honeybees has been reported previously.^34^ This low sequence divergence among LABs from honeybees can be explained as a result of adaptation of bacteria to a rich and variable content of carbohydrate as almost 50.0% of accessory genes coding proteins which are involved in carbohydrate metabolism and transport functions.^43^

In conclusion, it was revealed that PCR-RFLP could be used as a rapid and accurate technique for identification of LABs isolates from honeybees. Phylogenetic analysis showed that *Lactobacillus* and *Bifidobacteria* spp. isolated from honeybee belonged to different countries and were closely clustered with each other, fairly far from bacteria isolated from different sources such as dairy products.
